# The incidence of hypotension during general anesthesia: a single-center study at a university hospital

**DOI:** 10.1186/s40981-023-00617-9

**Published:** 2023-05-13

**Authors:** Nobuyuki Katori, Kentaro Yamakawa, Kotaro Kida, Yoshihiro Kimura, Shoko Fujioka, Tsunehisa Tsubokawa

**Affiliations:** Department of Anesthesiology, The Jikei University School of Medicine, 2-25-8 Nishishinbashi, Minato-Ku, Tokyo, 105-8461 Japan

**Keywords:** Intraoperative hypotension, Mean arterial blood pressure, General anesthesia, Gender, Vascular surgery, Emergency surgery

## Abstract

**Background:**

Although intraoperative hypotension (IOH) has been emerging as a serious concern during general anesthesia, the incidence of IOH has not been demonstrated clearly in the Japanese population.

**Methods:**

This single-center retrospective study investigated the incidence and the characteristics of IOH in non-cardiac surgery at a university hospital. IOH was defined as at least one fall of MAP during general anesthesia, which was categorized into the following groups: mild (65 to < 75 mmHg), moderate (55 to < 65 mmHg), severe (45 to < 55 mmHg), and very severe (< 45 mmHg). The incidence of IOH was calculated as a percentage of the number of events to the total anesthesia cases. Logistic regression analysis was performed to examine factors affecting IOH.

**Results:**

Eleven thousand two hundred ten cases out of 13,226 adult patients were included in the analysis. We found moderate to very severe hypotension occurred in 86.3% of the patients for at least 1 to 5 min, and 48.5% experienced severe or very severe hypotension. The results of the logistic regression analysis indicated female gender, vascular surgery, American Society of Anesthesiologists physical status classification (ASA-PS) 4 or 5 in emergency surgery, and the combination with the epidural block (EDB) were significant factors of IOH.

**Conclusions:**

IOH during general anesthesia was very frequent in the Japanese population. Female gender, vascular surgery, ASA-PA 4 or 5 in emergency surgery, and the combination with EDB were independent risk factors associated with IOH. However, the association with patient outcomes were not elucidated.

**Supplementary Information:**

The online version contains supplementary material available at 10.1186/s40981-023-00617-9.

## Background

Intraoperative hypotension (IOH) has been demonstrated as a critical concern that is associated with postoperative morbidities such as acute kidney injury, myocardial injury, and stroke [1–5]. The incidence of IOH varies among previous reports (16–93%) [1, 6, 7], which may be attributed to the use of different definitions and threshold values for IOH across the studies. The lack of a clear definition of IOH may contribute to the variability in reported incidence rates.

Recently, the Perioperative Quality Initiative published the consensus statement on intraoperative blood pressure, risk and outcomes for elective surgery; intraoperative mean arterial blood pressures below 60–70 mmHg are associated with postoperative morbidities including kidney and myocardial injury, and also death [8]. The workgroup also indicated not only the severity of hypotension but also the duration was associated with the morbidities. It is speculated that the relationship between IOH and patient outcomes indicated in the statement would be similar in Asia, but few data have been shown in a large population. In Japan, more than 3.5 million cases of non-cardiac surgery are performed under general anesthesia annually [9], however, even the incidence of intraoperative hypotension has not been demonstrated clearly in the Japanese population.

The aim of this study is to investigate the incidence and the characteristics of intraoperative hypotension in non-cardiac surgery at a Japanese university hospital.

## Methods

### Study design, setting, and participants

The protocol of this study was reviewed and approved by the institutional review board of the Jikei University School of Medicine (Tokyo, Japan) (approval number 31–160). The informed consent was waived because of this study's retrospective nature and anonymization of the data. Information on the conduct of the study and the protocol were posted on the website of the department and we guaranteed the opportunity of opt-out for the study subject. This single-institution retrospective observational study was performed in accordance with the Declaration of Helsinki and Clinical Trials Act established by the Japanese Ministry of Health, Labour and Welfare.

Patients who underwent surgery under general anesthesia in conjunction with or without additional regional anesthesia at the Jikei University Hospital between January 2018 and November 2019 were included in the study. Those younger than 20 years, undergoing cardiac surgery or cesarean section, or regional or neuraxial anesthesia combined with sedation, were excluded from the analysis. Only the first surgery was included in the analysis when the patients underwent multiple surgeries during the study period.

### Data collection

We extracted and analyzed the data from our electrical anesthesia records, which included biometric, medical, procedural, and physiological variables of patients undergoing general anesthesia between January 2018 and November 2019 at a 1075-bed university teaching hospital. Intraoperative hemodynamic data were obtained from the anesthesia record stored in the Philips Orsys® (Philips Japan, Tokyo, Japan) using the dedicated database software Vipros® (Philips Japan, Tokyo, Japan). Patient characteristics were extracted from a hospital information system (HOPE EGMAIN-GX, Fujitsu, Tokyo, Japan).

### Definition of intraoperative hypotension

Blood pressure was monitored from the beginning of anesthesia to the end of anesthesia by non-invasive and/or invasive measurements, which were recorded at an interval of ≤ 5 min (1, 2, 2.5, or 5 min) for non-invasive blood pressure (NIBP) and every minute for arterial blood pressure with vascular cannulation (ABP). When both NIBP and ABP were monitored, ABP data were adopted for analysis in preference. IOH was defined as at least one fall of MAP below the following threshold values between the beginning and end of surgery: 75, 70, 65, 60, 55, 50, 45, or 40 mmHg. The definition of IOH varies among the previous studies, which included systolic blood pressure, mean blood pressure, and percent change compared to baseline blood pressure measured before the induction of anesthesia, and the threshold values also vary [7]. We employed the absolute value of mean blood pressure as a primary outcome in this study because of its clinical utility, and the higher threshold for IOH was set at < 75 mmHg in order to examine the incidence of IOH broadly. The duration of hypotension for each hypotension threshold was calculated by Vipros® in each case. Because NIBP was measured at the interval of ≤ 5 min, the duration of hypotension for NIBP was calculated according to the measurement interval (1, 2, 2.5, or 5 min), which allowed for a 1 to 5-min error as described in the previous reports [2, 10, 11].

### Statistical analysis

IOH was defined as at least one fall of MAP below each hypotensive threshold and the incidence of IOH was calculated as a percentage of the number of cases with IOH to the total anesthesia cases. We also calculate the duration of every hypotensive event and examined the distribution of hypotension time for each hypotension threshold. Logistic regression analysis was performed with the occurrence of hypotensive events below each threshold as the dependent variable. Gender, age, height, weight, body mass index (BMI), American Society of Anesthesiologists Physical status classification (ASA-PS), emergency surgery, anesthetic method (with or without a combination of local anesthesia technique), anesthetic agent, surgical department, operation time, total input volume (fluid and blood transfusion), total output volume (urine volume and bleeding) and fluid balance (total input volume—total output volume) were used as independent variables. The variable was selected by the variable-increasing stepwise method based on the Wald test. Mann–Whitney U-test or Fisher’s exact test was performed to compare categorical data where applicable. The data were expressed as the number of events or cases and percentage, or mean with standard deviation where applicable. A *p*-value less than 0.05 was recognized as statistically significant.

## Results

A total of 13,226 adult patients underwent general anesthesia during the study period, among which 11,210 cases including 683 emergency cases were assigned to the analysis (Additional file 1: Appendix 1). The patient and surgical characteristics were shown in Table 1. There was no gender bias in the study population, and also no difference in average age and BMI between genders, although the mean height and weight were significantly larger in the male. Although 92.6% of the patients including emergency cases were classified as ASA-PS 1 or 2, the number of patients classified as ASA-PS 4 or 5 was significantly higher in the emergency surgery (Table 1). The most frequent preoperative comorbidity was hypertension (19.8%), followed by diabetes mellitus (9.2%) and renal dysfunction (5.7%) (Table 1).

**Table 1:** Patient and procedure characteristics (*n* = 11,210)

Category	Factors	number (per cent) or mean (± SD)	Overall average (± SD)
Demographic data	Gender: Male, Female	5753 (51.3%) 5457 (48.7%)	
	Age (years): Male, Female	57.0 (± 16.8) 54.7 (± 17.1)	55.9 (± 17.0)
	Height (cm): Male, Female	169.5 (± 6.8) * 156.6 (± 6.5)	163.2 (± 9.2)
	Weight (kg): Male, Female	68.5 (± 12.2) * 54.7 (± 10.6)	61.8 (± 13.4)
	BMI (kg/m^2^): Male, Female	23.8 (± 3.7) 22.3 (± 4.1)	23.1 (± 4.0)
	ASA-PS 1 1E	3579 (31.9%) 166 (1.5%)	
	2 2E	6289 (56.1%) 346 (3.0%)	
	3 3E	655 (5.8%) 157 (1.4%)	
	4 4E	4 (0.04%) 11 (0.1%)^#^	
	5 5E	0 3 (0.03%)^#^	
Comorbidities	Hypertension	2220 (19.8%)	
	Diabetes Mellitus	1034 (9.2%)	
	Angina Pectoris	309 (2.8%)	
	OMI	119 (1.1%)	
	Atrial Fibrillation	311 (2.8%)	
	Renal Dysfunction	638 (5.7%)	
	Hemodialysis	218 (1.9%)	
	Liver Dysfunction	199 (1.8%)	
	Cerebral Infarction	90 (0.8%)	
Surgical department	Otolaryngology	2691 (24.0%)	
	Orthopedics	1668 (14.9%)	
	Gynecology/Obstetrics	1238 (11.0%)	
	Urology	799 (7.1%)	
	Lower intestinal surgery	656 (5.9%)	
	Liver surgery	611 (5.5%)	
	Plastic surgery	523 (4.7%)	
	Upper intestinal surgery	496 (4.4%)	
	Neurosurgery	453 (4.0%)	
	Respiratory surgery	451 (4.0%)	
	Vascular surgery	449 (4.0%)	
	Breast, thyroid surgery	439 (3.9%)	
	Others	736 (6.6%)	
Anesthesia	GA	8793 (78.4%)	
	GA + EDB	1497 (13.4%)	
	GA + PNB	920 (8.2%)	
Anesthetic agent	Desflurane	9266 (82.7%)	
	Sevoflurane	659 (5.9%)	
	Propofol (TIVA)	1042 (9.3%)	
	Others	243 (2.1%)	
Operation time (min)			164 (± 121)
Anesthesia time (min)			226 (± 134)
Total input (ml)			1452 (± 1397)
(ml/hour)			380 (± 323)
	Crystalloid (ml)		1305 (± 1056)
	Colloid (ml)		94 (± 270)
	Transfusion (ml)		40 (± 378)
Total output (ml)			466 (± 813)
	Blood loss (ml)		114 (± 394)
	Urine (ml)		305 (± 484)
Fluid balance (ml)			+ 1338 (± 1204)

Renal dysfunction was defined as estimated glomerular filtration rate < 60 ml/min/1.73m^2^

Liver dysfunction was defined by the elevation of liver enzymes or based on clinical diagnosis

*BMI* Body mass index, *ASA-PS* American Society of Anesthesiologists Physical Status, *OMI* Old myocardial infarction, *GA* General anesthesia, *EDB* Epidural block, *PNB* Peripheral nerve block, *TIVA* Total intravenous anesthesia

^*^p < 0.05 compared to the female

^#^*p* < 0.01 compared to the elective surgery

**Fig. 1:**
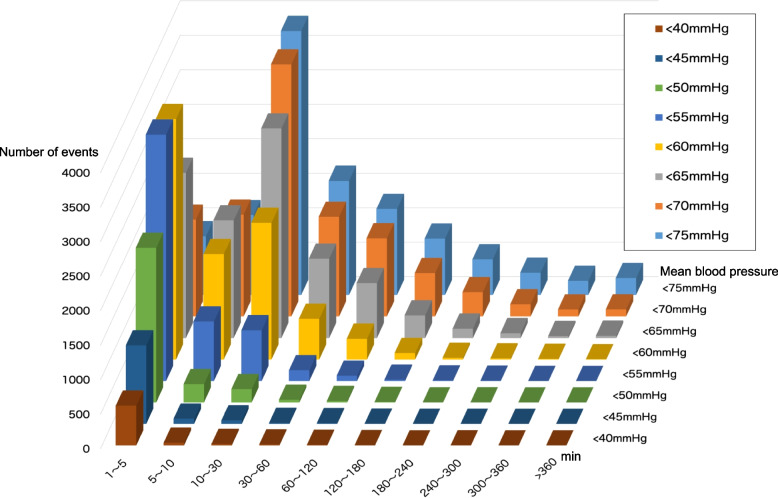
Number of cases from the relation between each hypotension level and the duration of hypotension

The incidences of IOH at each hypotension threshold were as followed:96.8% for < 75 mmHg, 93.5% for < 70 mmHg, 86.3% for < 65 mmHg, 71.7% for < 60 mmHg, 48.5% for < 55 mmHg, 24.7% for < 50 mmHg, 11.6% for < 45 mmHg, and 5.9% for < 40 mmHg. The distribution of hypotension time for each hypotension threshold was shown in Fig. 1. We also calculated the percentage of events that continued for more than or equal to 10 minutes at each hypotensive threshold: 81.4% for < 75 mmHg, 57.6% for < 65 mmHg, 18.4% for 55 mmHg, and 6.5% for < 45 mmHg, respectively. Hence, the incidence of IOH continuing for ≥ 10 minutes in each hypotensive threshold described above was 78.9%, 49.7%, 8.9%, and 0.8%, respectively.

**Fig. 2:**
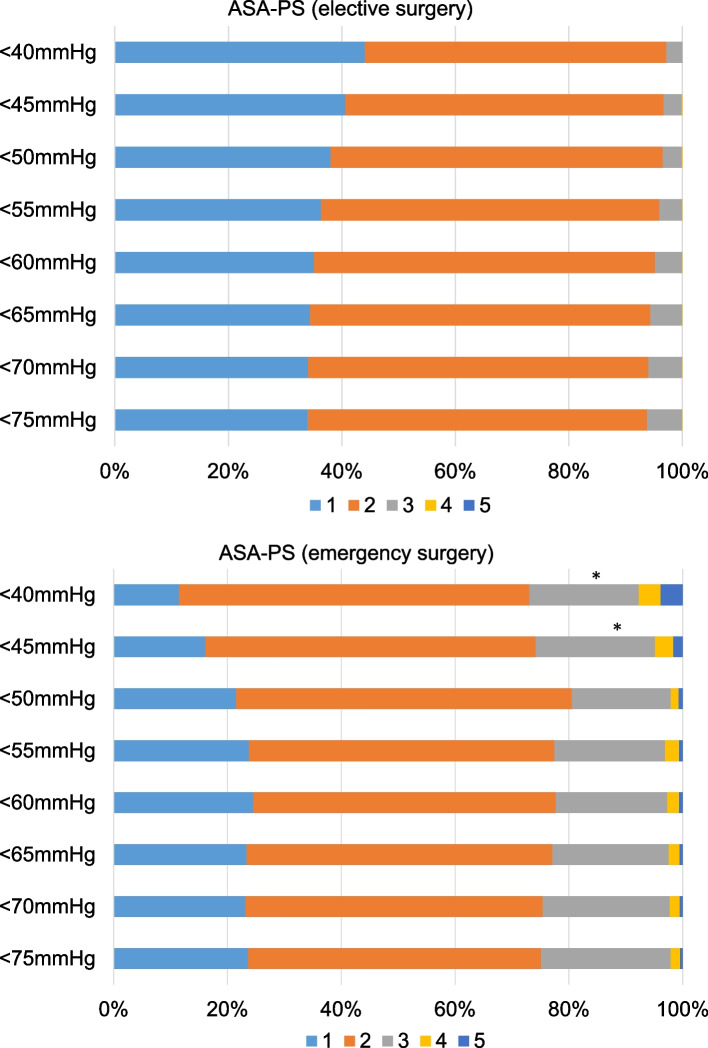
ASA-PS distribution in each hypotension threshold (**A**) elective surgery, (**B**) emergency surgery. ASA-PS 4 and 5 were a risk factor for very severe hypotension in the emergency surgery (*p* < 0.001)

**Fig. 3:**
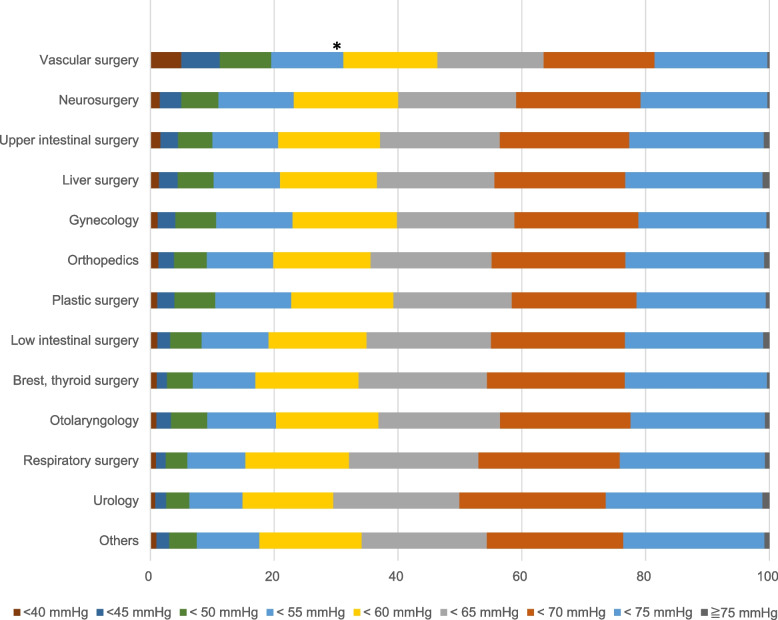
Hypotensive events by departments. Hypotension less than 55 mmHg was significantly frequent in vascular surgery (*p* < 0.01)

The results of logistic regression analysis indicated the female was a significant factor for IOH in the hypotension thresholds between 50 and 75 mmHg (*p* < 0.05), although it was not for hypotension < 45 mmHg. The relative risks for each hypotensive threshold compared to the male were as followed: 1.64 (95%CI: 1.51–1.78) for < 75 mmHg, 1.50 (95%CI: 1.38–1.61) for < 70 mmHg, 1.40 (95%CI: 1.32–1.47) for < 65 mmHg, 1.36 (95%CI: 1.29–1.44) for < 60 mmHg, 1.29 (95%CI: 1.19–1.38) for < 55 mmHg, 1.22 (95%CI: 1.17–1.27) for < 50 mmHg, 1.07 (95%CI: 0.98–1.13) for < 45 mmHg, and 1.00 (95%CI: 0.97–1.02) for < 40 mmHg. ASA-PS 4 and 5 were risk factors for hypotension < 45 mmHg in emergency surgery (*p* < 0.001) (Fig. 2). Preoperative comorbidities were not generally significant factors for IOH, although hypertension was related to an increased risk of IOH < 40 mmHg (relative risk: 1.06, 95%CI: 1.04–1.09). Renal dysfunction was also a significant factor for IOH < 45 mmHg (relative risk: 1.03, 95%CI: 1.01–1.05) and 40 mmHg (relative risk: 1.04, 95%CI: 1.02–1.06). Concerning anesthesia, the combination with EDB was a significant factor for IOH in all thresholds, although the variety of anesthetic agents was not (data not shown). The relative risks of EDB for IOH in each hypotensive threshold were as followed: 1.08 (95%CI: 1.05–1.11) for < 75 mmHg, 1.10 (95%CI: 1.08–1.13) for < 70 mmHg, 1.11 (95%CI:1.09–1.13) for < 65 mmHg, 1.12 (95%CI: 1.10–1.13) for < 60 mmHg, 1.11 (95%CI: 1.09–1.12) for < 55 mmHg, 1.12 (95%CI: 1.09–1.14) for < 50 mmHg, 1.14 (95%CI: 1.11–1.18) for < 45 mmHg, and 1.12 (95%CI: 1.07–1.16) for < 40 mmHg. Among surgical departments, vascular surgery was the only significant factor for hypotension < 55 mmHg (Fig. 3).

## Discussion

This is the first large-scale report that investigated the incidence of IOH in Japan. In this single-center retrospective study, we found that IOH occurred very frequently during general anesthesia, which depends on the definition of the hypotension threshold. As an overall trend, severe hypotension (MAP < 55 mmHg) occurred frequently (48.5% of the study population) although the duration of hypotension was relatively short. However, the impact of IOH on postoperative mortality and morbidities is associated not only with the degree of hypotension but also with its duration [8]. A previous report indicated hypotension of MAP < 55 mmHg has been indicated to increase the risk of mortality and organ injury when continued for more than 5 to 10 min [12]. It also described that even mild to moderate hypotension (55 ≤ MAP < 75 mmHg) could increase the risk of organ damage including acute kidney injury and myocardial damage with the duration of hypotension [12]. In the present study, 26% of hypotension < 55 mmHg continued for more than 5 min and 14% for more than 10 min, indicating that severe hypotension which could significantly increase the risk of mortality and organ injury, occurred frequently. Meanwhile, the results of our study indicated a trend that the duration of hypotension was prolonged as the degree of hypotension became less severe (Fig. 1). Hemodynamic management varying in attending anesthesiologists might have influenced the prolonged duration of mild to moderate hypotension in this study, despite the avoidance of MAP less than 65 mmHg being recognized as a general concept of hemodynamic management in our institute.

The results of logistic regression analysis indicated significant risk factors for IOH: female gender, vascular surgery, ASA-PS 4 or 5 in emergency surgery, and the combination EDB. It has been reported that female hormones affect the sensitivity to the cardiovagal reflex, which may contribute to the gender difference in hypertension [13]. However, it is difficult to discuss the effect of hormones on IOH based on the results of our study. Among surgical departments, vascular surgery was identified as a risk factor for severe IOH. Patients who receive vascular surgery are generally elder and have higher pulse pressure and isolated systolic hypertension due to reduced arterial compliance resulting from increased arterial wall stiffness [14, 15]. The increase in pulse pressure due to increased arterial wall stiffness is an independent factor defining sympathetic baroreflex sensitivity in the elderly [16], and can lead to blood pressure fluctuations due to impaired baroreflex sensitivity [17]. Thus, it could be speculated vascular surgery patients are more prone to IOH due to arterial wall stiffness [18]. On the other hand, the presence of preoperative hypertension was not a risk factor for IOH but rather reduced the risk of hypotension ranging between 45 and 75 mmHg in the present study. As hypertension results from various etiology and pathophysiology, hypertension is not always associated with impaired baroreflex sensitivity due to arterial wall stiffness; hence, the presence of hypertension might not always contribute to the development of IOH. ASA-PS 4 or 5 was also indicated as a risk factor for IOH < 45 mmHg in emergency surgery. As the patients in this physical status classification are generally suffering from severe cardiovascular diseases including unstable ischemic heart disease, acute or chronic heart failure, and ruptured aortic aneurysm, they might be prone to severe hypotension due to poor preoperative hemodynamic reserve, especially in emergency surgery because of insufficient preoperative patient evaluation and management.

The mechanism of hypotension induced by EDB is well-established [19]. A retrospective study, in which post-induction hypotension and early IOH were examined, indicated epidural anesthesia was an independent risk factor for early IOH [20]. Although we did not examine the timing of hypotension in this study, we consider the results of our study to be consistent with the previous study.

This retrospective study has several limitations. The incidence of IOH was relatively higher than those previously reported, however, we could not indicate how this high incidence of IOH affected adverse clinical outcomes including postoperative mortality or organ injury because the aim of the study was to investigate the incidence and clinical features of IOH in the Japanese population. The second limitation is that the present study did not examine the use of vasopressors or inotropes during the surgery. Therefore, it is difficult to distinguish whether the hypotension in this study occurred despite therapeutic interventions or as a result of the acceptance by the attending anesthesiologist without intervention, although hypotensive anesthesia is not employed in our institute. Because this was s retrospective study, therapeutic interventions including the use of vasoactive agents were at the discretion of the attending anesthesiologist. The results of this study might imply that adherence to avoiding IOH was not always respected among anesthesiologists. Finally, we could not conclude the results of this study would reflect the situation of IOH in Japan because this was a single-center study, although this is the first large-scale study that included more than 10 thousand patients. A future multi-center study may shed more light on the actual situation of IOH in Japan.

## Conclusions

This is the first report that indicates the incidence of IOH during general anesthesia in non-cardiac surgery in a large Japanese population.

IOH during general anesthesia frequently occurred in this single-center retrospective study. IOH < 65 mmHg continued at least for 1–5 min in 86.3% of patients and for ≥ 10 min in almost 50% of patients. Female gender, vascular surgery, ASA-PA 4 or 5 in emergency surgery, and the combination of EDB with general anesthesia were independent risk factors associated with IOH, although the patient outcome including mortality or morbidities is not elucidated in this study.

## Supplementary Information

**Additional file 1: Appendix 1.** Study flow diagram.

## Data Availability

The datasets generated and/or analyzed during the current study are not publicly available because the institutional rules strictly prohibit releasing native data on the web but are available from the corresponding author upon a reasonable request.

## Abbreviations

IOH: Intraoperative hypotension
MAP: Mean arterial pressure
NIBP: Noninvasive blood pressure
ABP: Arterial blood pressure
BMI: Body mass index
ASA-PS: American Society of Anesthesiologists Physical Status
EDB: Epidural block
PNB: Peripheral nerve block
