# Correction: The incidence of hypotension during general anesthesia: a single-center study at a university hospital

**DOI:** 10.1186/s40981-023-00624-w

**Published:** 2023-06-01

**Authors:** Nobuyuki Katori, Kentaro Yamakawa, Kotaro Kida, Yoshihiro Kimura, Shoko Fujioka, Tsunehisa Tsubokawa

**Affiliations:** Department of Anesthesiology, The Jikei University School of Medicine, 2‑25‑8 Nishishinbashi, Minato‑Ku, Tokyo, 105‑8461 Japan

**Correction: JA Clin Rep 9, 23 (2023)**

**https://doi.org/10.1186/s40981-023-00617-9**

Following publication of the original article [1], the author reported that there are citations to Figs. 2 and 3 that should have not been included in the second paragraph of the 'Results' section. The paragraph should read:

From

We also calculated the percentage of events that continued for more than or equal to 10 min at each hypotensive threshold using the data presented in Fig. 3. The percentage of hypotensive events that continued for more than or equal to 10 min at < 75 mmHg, < 65 mmHg, < 55 mmHg, and < 45 mmHg was 81.4%, 57.6%, 18.4%, and 6.5%, respectively. Considering the incidence of IOH shown in Fig. 2, the incidence of IOH continuing for ≥ 10 min in each hypotensive threshold described above was 78.9%, 49.7%, 8.9%, and 0.8%, respectively.

To

We also calculated the percentage of events that continued for more than or equal to 10 min at each hypotensive threshold: 81.4% for < 75 mmHg, 57.6% for < 65 mmHg, 18.4% for 55 mmHg, and 6.5% for < 45 mmHg, respectively. Hence, the incidence of IOH continuing for ≥ 10 min in each hypotensive threshold described above was 78.9%, 49.7%, 8.9%, and 0.8%, respectively.

The original article has been updated.
