# Supplementary material for: The incidence of hypotension during general anesthesia: a single-center study at a university hospital
Source: JA Clin Rep. 2023 May 13;9:23. doi: 10.1186/s40981-023-00617-9 (PMC10182227; doi:10.1186/s40981-023-00617-9)
Supplement: Supplementary file 1 — Additional file 1: Appendix 1. Study flow diagram. [file 40981_2023_617_MOESM1_ESM.pptx]

## Slide 1
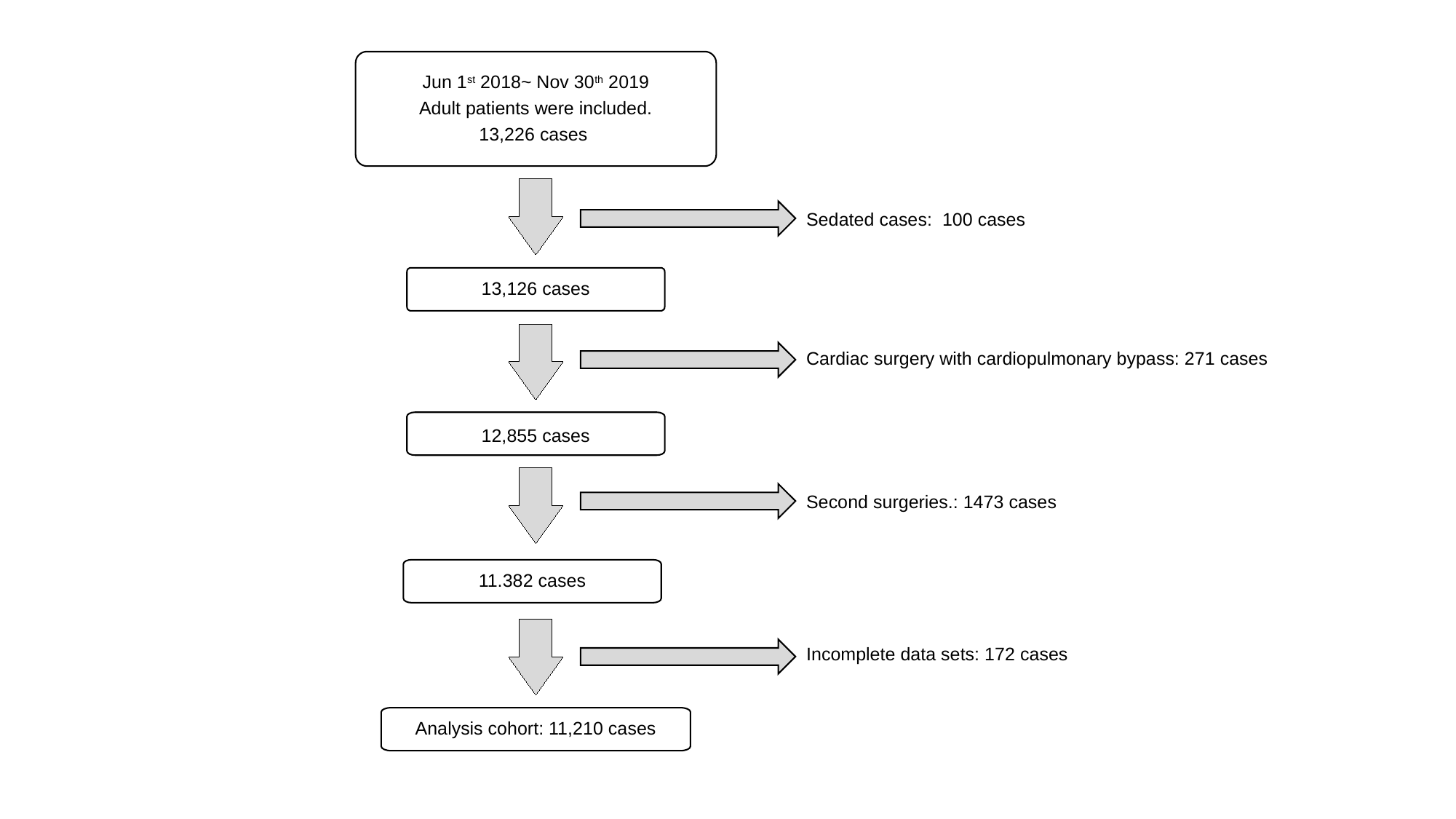

Jun 1st 2018~ Nov 30th 2019
Adult patients were included.
13,226 cases
Sedated cases: 100 cases
13,126 cases
Cardiac surgery with cardiopulmonary bypass: 271 cases
12,855 cases
Second surgeries.: 1473 cases
11.382 cases
Incomplete data sets: 172 cases
Analysis cohort: 11,210 cases
